# Synergistic Regulation of Combustion Behavior and Safety Characteristics of Graphene Modified Core–Shell Al@AP Composites

**DOI:** 10.3390/nano15110853

**Published:** 2025-06-02

**Authors:** Jiahui Shi, Jiahao Liang, Xiaole Sun, Yingjun Li, Haijun Zhang, Xueyong Guo, Shi Yan, Junwei Li, Jianxin Nie

**Affiliations:** 1 State Key Laboratory of Explosion Science and Safety Protection, Beijing Institute of Technology, Beijing 100081, China; 2 School of Aerospace Engineering, Beijing Institute of Technology, Beijing 100081, China; 3 Chongqing Hongyu Precision Industry Co., Ltd., Chongqing 402760, China; 4 601 Institute, The Sixth Academy of China Aerospace Science and Industry Corporation, Hohhot 010076, China; 5 Xi’an Modern Control Technology Research Institute, Xi’an 710065, China

**Keywords:** al@ammonium perchlorate, graphene, thermal decomposition characteristics, combustion performance, safety performance

## Abstract

Improving the energy release and safety of composite solid propellants is a key focus in energetic materials research. Graphene, with its excellent thermal conductivity and lubrication properties, is a promising additive. In this study, Al@AP core–shell particles doped with graphene were prepared via an in-situ deposition method. The structure, thermal decomposition, combustion, and safety performance of the graphene-doped Al@AP samples were investigated. Results showed that AP effectively coated aluminium to form a typical core-shell structure, with graphene uniformly loaded into the framework. Graphene contents of 1.0 and 4.0 wt.% reduced AP’s thermal decomposition temperature by 0.97 and 16.68 °C, respectively. Closed-bomb and laser ignition tests revealed that pressure rise rates and combustion intensity increased with graphene content up to 1.0 wt.% but declined beyond that. Peak pressure reached 114.65 kPa at 1.0 wt.% graphene, and the maximum pressure increase rate was 13.29 kPa ms^−1^ at 2.0 wt.%. Additionally, graphene significantly improved safety by reducing sensitivity to impact and friction. The enhanced performance is attributed to graphene’s large surface area and excellent thermal and electrical conductivity that promote AP decomposition and combustion, combined with its lubricating effect that enhances safety, though excessive graphene may hinder these benefits. This study provides balanced design criteria for graphene-doped Al@AP as solid propellants.

## 1. Introduction

Solid propellants provide propulsion energy for aerospace systems, and their rapid energy release and safety performance are actively researched [1]. Typical composite solid propellants are composed of oxidisers (for example, ammonium perchlorate (AP) and ammonium nitrate), fuels (aluminium powder), and polymer binders [2,3]. The oxidiser constitutes the largest proportion (typically >60%), indicating its significant effect on the performance of solid propellants [4]. Al@ammonium perchlorate (Al@AP, AP = [NH_4_ClO_4_]) remains the primary oxidiser in solid propellants because of its high density, oxygen content, heat release, gas production, excellent stability, and low cost [5,6]. Owing to its high energy density, high combustion heat value, and low cost, Al powder is a major fuel in solid propellants, which significantly increases the combustion temperature and theoretical specific impulse [7,8,9,10]. The large proportions of AP and Al powder in typical solid propellant formulations significantly influence the combustion and safety performance of propellants, while core-shell structures exhibit improved ignition performance [11]. Furthermore, recent studies have shown that coating Al with AP to form a core-shell structure is effective for enhancing combustion performance. Therefore, investigating the combustion process and safety performance of Al@AP core-shell composites is crucial.

The burn rates of solid propellants are often regulated by adding catalysts [12,13,14]. Common catalysts used to increase the burn rate include metal oxides, nano-catalysts, and ferrocene derivatives [15,16,17]; however, the addition of catalysts may significantly increase the impact and/or friction sensitivity of a solid propellant, which can pose safety issues during production, storage, and use [18].

Over the past decade, two-dimensional materials such as graphene have been used extensively in various fields. Graphene is a novel nanocarbon material with a two-dimensional honeycomb crystal structure that exhibits superior physical, chemical, and mechanical properties compared to traditional carbonaceous materials [19]. Graphene can act as an effective catalyst in chemical reactions because of its excellent thermal and electrical conductivities and exceptionally large specific surface area, thereby facilitating the thermal decomposition of energetic materials [20]. Consequently, graphene-based materials are widely used in the field of energetic materials [21]. In addition, the excellent lubrication properties of graphene significantly enhance the safety performance of energetic materials. Although graphene-based materials have been investigated in the field of energetic materials, the mechanism by which pure graphene affects the thermal decomposition, ignition, and combustion and safety performance of the AP oxidiser remains unclear; understanding this mechanism is critical for the application of functional graphene materials in solid propellants. Previous studies have revealed an opposing relationship between reducing the decomposition temperature of energetic materials and improving safety performance [22,23]; however, methods to balance the burn rate and safety characteristics of energetic materials remain scarce.

In this study, Al@AP samples were prepared using an in-situ deposition method and uniformly doped with graphene. The morphology and composition of the samples were analysed using scanning electron microscopy (SEM) and X-ray diffraction (XRD). The effect of graphene content on the thermal decomposition characteristics and combustion performance of the samples was subsequently investigated using thermogravimetric analysis-differential scanning calorimetry-Fourier-transform infrared spectroscopy (TG-DSC-FTIR), laser ignition, and closed-bomb testing. The safety performance of the samples was examined using the BAM method for sensitivity testing. Finally, the mechanism by which graphene affects the combustion characteristics and safety performance of the samples was analysed. These findings provide valuable insights into the application of graphene-based materials in solid propellants.

## 2. Materials and Methods

### 2.1. Preparation of Samples

**Figure 1:**
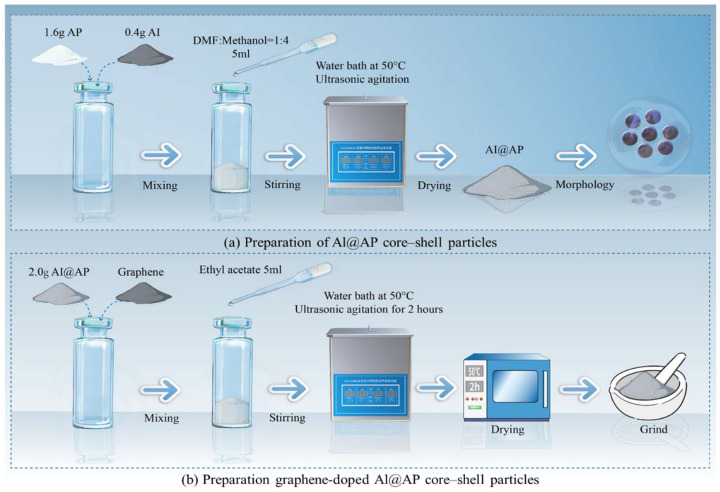
Hierarchical fabrication process of graphene-doped Al@AP core-shell composites.

The details of the materials used in this study are listed in Table 1. The preparative process of the Al@AP composite particles is shown in Figure 1a. The specified masses of AP and Al listed in Table 2 were weighed and placed in a vial, to which a 5 mL mixture of DMF and methanol (1:4 *v*/*v*) was added. Next, the mixture was dispersed and dried using ultrasonic agitation in a water bath at 50 °C to obtain an Al@AP core-shell material. The process of preparing the graphene-doped Al@AP samples is illustrated in Figure 1b. The prepared Al@AP and various masses of graphene were placed in a vial, followed by the addition of 5 mL of ethyl acetate. The mixture was then dispersed using ultrasonic agitation in a water bath at 50 °C for 2 h. Subsequently, the samples were dried in an oven at 50 °C and ground using an agate mortar.

### 2.2. Morphological and Structural Characterisation

Transmission electron microscopy (TEM; JEM-F200, Japan) was used to analyse the coating state of the Al@AP core-shell structure. SEM (Hitachi SU8600, Japan) was used to examine the microstructures of the graphene-doped Al@AP samples. XRD was employed to investigate the phase composition of the samples in the scanning range of 2θ = 5–90°.

### 2.3. Thermal Decomposition Characterisation

Thermogravimetric analysis was conducted using a simultaneous thermal analyser-infrared mass spectrometer (Netzsch-STA449F3, Germany; FTIR Nicolet iS20, America; Netzsch-QMS 403, Germany, accessed from www.eceshi.com). Approximately 1.0 mg of sample was heated within the range of 40–800 °C under ambient conditions at a heating rate of 10 °C min^−1^. High-purity Ar gas (flow rate = 240 mL min^–1^) was used for purging.

### 2.4. Laser Ignition Test for Characterising the Combustion Characteristics

**Figure 2:**
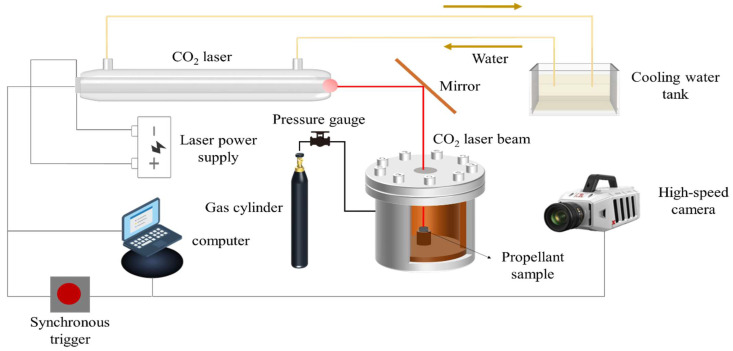
Schematic of the CO_2_ laser ignition combustion test system.

The combustion characteristics of the samples were analysed using a CO_2_ laser ignition system with an output power of 10 W and a duration of 200 ms. A high-speed photography camera (Revealer X113; Hefei Agile Device Co., Ltd., China) with a frame rate of 8000 frames per second and an exposure time of 30 μs was used to analyse the ignition properties, combustion processes and flame morphology of the samples. The experimental setup in which the combustion process was recorded in synchronised triggering mode is displayed in Figure 2.

### 2.5. Closed-Bomb Testing

**Figure 3:**
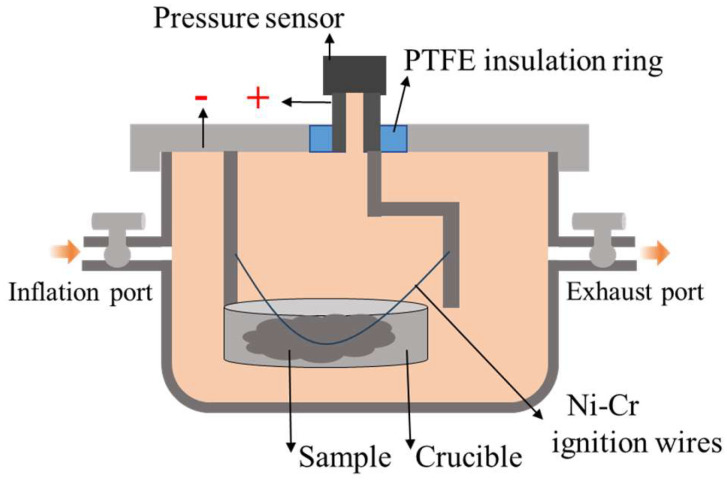
Schematic of the closed-bomb test device.

A schematic of the experimental test device used for obtaining the combustion pressure-time curves is shown in Figure 3. During the experiment, 30 ± 0.1 mg samples were weighed and placed into a 50 mL closed-bomb vessel with an electric ignition controller.

### 2.6. Impact and Friction Sensitivity Testing

Safety is critical for energetic materials. The impact (BFH-12, IDEA SCIENCE Group) and friction sensitivities (FSA-12, IDEA SCIENCE Group) were tested using the standard BAM method.

## 3. Results and Discussion

### 3.1. Surface Morphology and Structural Analysis

**Figure 4:**
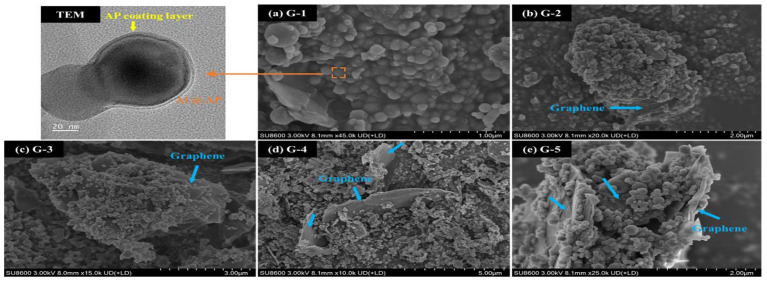
Microstructural evolution of graphene-doped Al@AP composites with varied doping concentrations.

SEM was used to examine the microstructure of the samples and analyse the distribution and morphology of their components. As shown in Figure 4, sample G-1 exhibited a uniform distribution of Al@AP on the graphene surface. Some particles exhibited aggregation because of the recrystallisation of AP; however, no large aggregates were observed. The TEM image indicated approximately 5-nm-thick AP layers were present on the surface of the Al particles, which is suggestive of an effective coating and typical core-shell structure. Owing to the addition of graphene at a low concentration and considering the large specific surface area of graphene in G-2, the Al@AP particles were distributed on the surface of graphene with graphene wrapped around them. In G-3, more graphene was embedded in the Al@AP particles due to a higher graphene content. However, in G-4, with a graphene content of 2 wt.%, graphene was wrapped around numerous Al@AP particles. Finally, all the Al@AP particles were distributed on the graphene sheets in G-5. Overall, the distribution of Al@AP varied with increasing graphene content. Initially, the Al@AP particles were partially coated with graphene; however, the Al@AP particles tended to envelope with increasing graphene content. This transition may influence the combustion behaviour of Al@AP particles.

**Figure 5:**
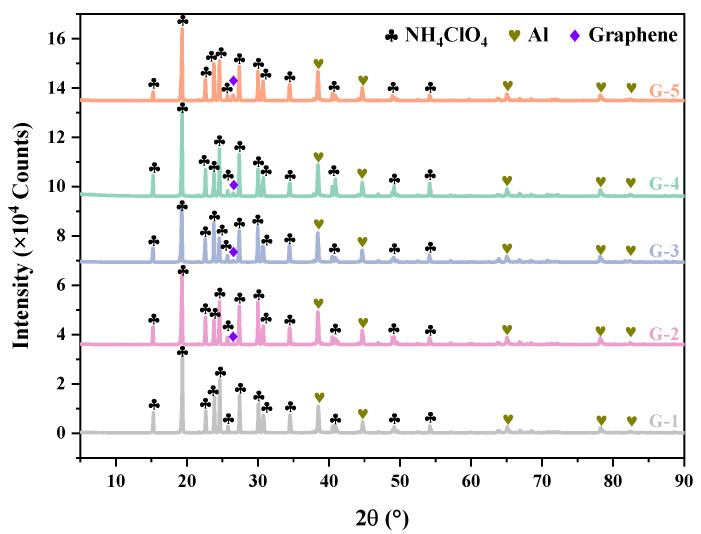
XRD patterns of graphene-doped Al@AP composites with varied doping concentrations.

The XRD patterns of the Al@AP samples with varying graphene contents are shown in Figure 5. The diffraction peak at 2θ = 26.49° is attributed to the (002) plane of graphene. The peaks at 2θ = 38.50°, 44.72°, 65.10°, 78.22°, and 82.45° were assigned to the diffraction peaks of face-centred cubic Al, and represent the (111), (200), (220), (311), and (222) crystallographic planes, respectively. The peaks at 2θ = 19.32°, 24.58°, 30.72°, 34.39°, and 63.95° were assigned to the diffraction peaks of AP. The XRD patterns showed sharp diffraction peaks for all samples, indicating their high crystallinity and the absence of amorphous phases. The samples prepared using the in-situ deposition method exhibited clear diffraction peaks, suggesting that this method promoted AP crystallisation without significantly altering the crystalline structure of the raw materials. In addition, the crystalline peaks of each component were distinctly identifiable in the diffraction patterns, providing an indication of the effective loading of graphene into the Al@AP framework.

### 3.2. Analysis of the Thermal Decomposition Characteristics

**Figure 6:**
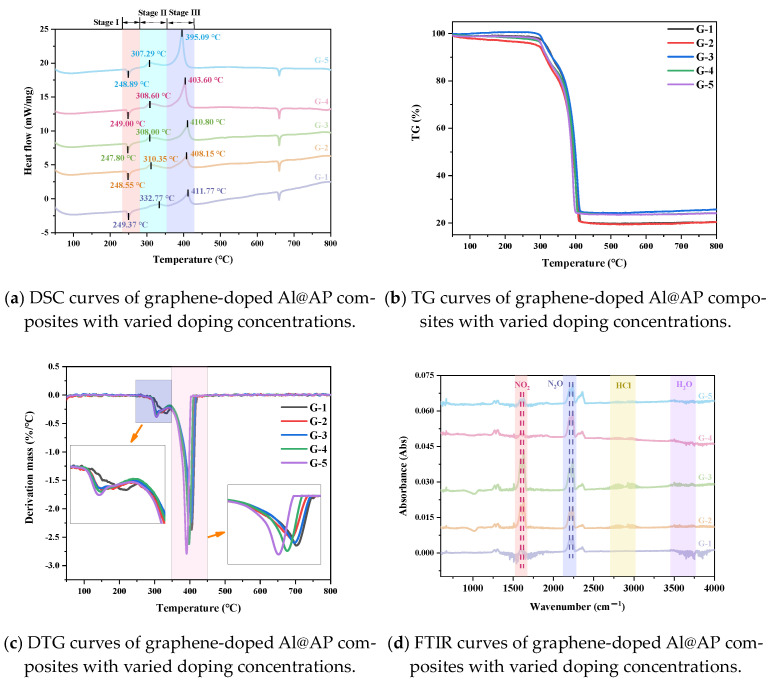
(**a**) DSC, (**b**) TG, (**c**) DTG, and (**d**) FTIR curves for samples G-1 to G-5.

The TG-DSC-FTIR traces of the Al@AP samples with varying graphene contents are shown in Figure 6. The results show that graphene significantly influences the thermal reaction characteristics of the Al@AP samples. The DSC traces shown in Figure 6a revealed that G-1 (Al@AP) gives rise to four characteristic peaks. The first stage at 249.37 °C represents an endothermic phase transition owing to the transition from an orthorhombic to a cubic crystalline structure [24]. The second stage shows an exothermic process at 332.77 °C, which corresponds to the low-temperature decomposition (LTD) of AP, involving solid-gas reactions, dissociation, and sublimation processes. The third stage corresponds to the high-temperature decomposition (HTD) of AP, in which gas-phase reactions are the controlling steps. AP fully decomposes into volatile products such as NO, O_2_, Cl_2_, and H_2_O, with an HTD of 411.77 °C. Compared with pure Al@AP, the addition of graphene does not affect the crystalline transition process of AP; however, it decreases both the LTD and HTD temperatures. When 0.5, 1.0, 2.0, and 4.0 wt.% graphene was added, the LTD temperature decreased to 310.35, 308.00, 308.60, and 307.29 °C, respectively, exhibiting reductions of 22.42, 24.77, 24.17, and 25.48 °C, respectively. The HTD temperatures of G-2 to G-5 were 408.15, 410.80, 403.60, and 395.09 °C, respectively, exhibiting reductions of 3.62, 0.97, 8.17, and 16.68 °C, respectively. These results are consistent with those of previous studies, in which graphene addition decreased the HTD temperature of AP [25,26]. A decrease in the HTD temperature implies an increase in the combustion rate of the samples [27]. Therefore, graphene effectively promotes the thermal decomposition of AP, and the effect is enhanced with increasing graphene content. This enhancement is attributed to the excellent electrical and thermal conductivities of graphene, which facilitate electron and heat transfer during AP decomposition.

The TG and DTG traces of the samples are shown in Figure 6b,c, respectively. The decomposition process of the pure Al@AP sample (G-1) occurred in two stages; thermogravimetric analysis indicated a mass loss of 14.92% after the first decomposition stage. Both the initial weight loss temperature and peak temperatures of the two DTG stages decreased with increasing graphene content, suggesting that graphene promoted the thermal decomposition of Al@AP, with a more pronounced effect observed at higher graphene loadings. This phenomenon can be attributed to the large specific surface area of graphene, which facilitates the adsorption of Al@AP particles to graphene. The efficient electronic transfer ability of graphene leads to the adsorption of NH_3_ and HClO_4_, generated during AP thermal decomposition, onto the graphene surface, thereby delaying their entry into the gas phase and their participation in oxidation reactions. This results in a decrease in the decomposition temperature of AP.

The FTIR spectra of the gas-phase products at the peak weight loss temperatures for samples G-1 to G-5 indicate the presence of N_2_O (2238 and 2201 cm^−1^), NO_2_ (1630 and 1598 cm^−1^), H_2_O (3500–4000 cm^−1^), and HCl (2700–3012 cm^−1^; Figure 6d, and indicates that the main gaseous products formed during the thermal decomposition of AP were N_2_O and NO_2_. For G-1, the absorption intensity of N_2_O was higher than that of NO_2_. With increasing graphene content in G-2 and G-3, the absorption intensity of NO_2_ was greater than that of N_2_O. A further increase in the graphene content resulted in the absorption intensity of N_2_O exceeding that of NO_2_ (see G-4 and G-5). The variations in the N_2_O and NO_2_ intensities suggest a competitive relationship in their formation during the thermal decomposition of AP. Therefore, the addition of graphene significantly influences the catalytic activity of AP and its thermal decomposition, and to varying degrees, based on the amount of graphene added.

The initial step in the thermal decomposition of AP (Equation (1)) involves proton transfer, in which *NH*_4_*ClO*_4_ dissociates to form adsorbed *NH*_3_ and *HClO*_4_. Next, during the LTD process, reactions occur between *NH*_3_ molecules adsorbed onto the particle surface; *NH*_3_ remains adsorbed on the particle surface without oxidation by *HClO*_4_ decomposition products at lower temperatures, resulting in eventual complete AP surface coverage by *NH*_3_, thus concluding LTD [28,29].

(1) $${NH}_{4}{ClO}_{4}↔{NH}_{3}-H-{ClO}_{4}↔{NH}_{3}-{HClO}_{4}↔{NH}_{3}\left(aq\right)+{HClO}_{4}\left(aq\right)↔{NH}_{3}\left(g\right)+{HClO}_{4}\left(g\right)$$

The HTD of AP involves gas-phase reactions, in which the adsorbed *NH_3_* and *HClO_4_* undergo desorption into the gas phase. In the gas phase, *HClO_4_* further decomposes to generate oxidised products that then oxidise NH_3_ to provide the final products, such as *HCl*, *H_2_O*, *N_2_O*, *NH_3_*, and *NO_2_* [30].

(2) $${4HClO}_{4}\to {Cl}_{2}+{5O}_{2}+{2ClO}_{2}+{2H}_{2}O$$

(3) $${2Cl}_{2}+{2H}_{2}O\to 4HCl+O_{2}$$

(4) $$2{NH}_{3}+{2ClO}_{2}\to N_{2}O+{Cl}_{2}+{3H}_{2}O$$

(5) $$N_{2}O+O_{2}\to NO+{NO}_{2}$$

According to the generally accepted theory for AP thermal decomposition, *NH_3_* reacts with *ClO_2_* to form *N_2_O*, which is further oxidised to *NO_2_*. In G-2 and G-3, higher *NO_2_* content is indicative of more complete AP decomposition, suggesting that graphene facilitates more thorough AP decomposition. In contrast, G-4 and G-5 yielded more *N_2_O*, indicative of incomplete decomposition caused by residual *O_2_*. The SEM images revealed that moderate graphene doping increases the contact area between graphene and Al@AP, facilitating *NH_4_ClO_4_* dissociation, providing abundant active sites for AP decomposition and reducing decomposition temperatures. However, excessive graphene encapsulation around the Al@AP particles in G-4 and G-5 formed closed spaces that inhibit AP decomposition, resulting in incomplete decomposition. Such variations in thermal decomposition inevitably affect the combustion characteristics of the samples.

### 3.3. Analysis of Closed Combustion Pressure

**Figure 7:**
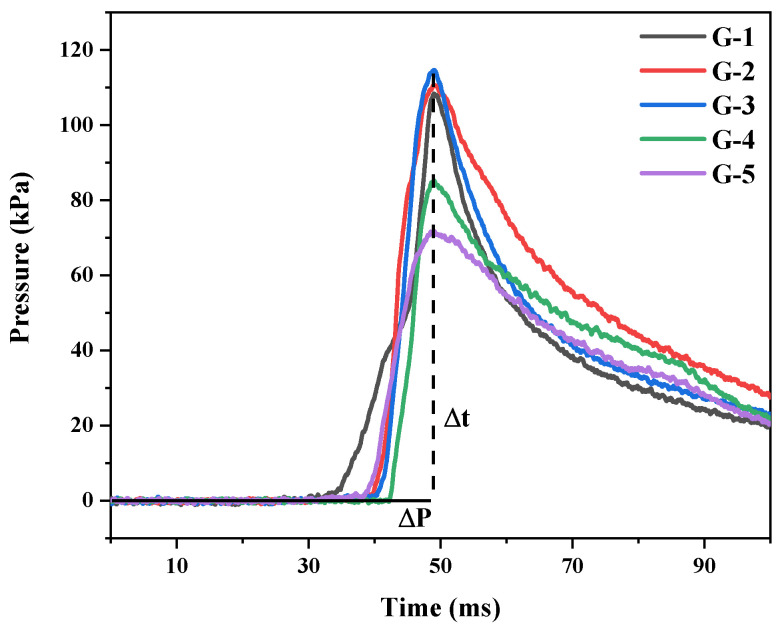
Pressure-time curves of graphene-doped Al@AP composites with varied doping concentrations.

The pressure-time curves of Al@AP with different graphene loadings are shown in Figure 7, and Table 3 lists the calculated parameters. The peak pressure and rate of pressure increase of the samples increased when the graphene content was increased from 0 (G-1) to 1.0 wt.% (G-3); the peak pressure increased by 2.27 and 6.30 kPa upon the addition of 0.5 and 1.0 wt.% graphene, respectively, and the rate of pressure increase correspondingly increased by 6.23 and 7.83 kPa ms^−1^, respectively. However, both parameters for the samples containing 2.0 (G-4) and 4.0 wt.% graphene (G-5) were lower than those of G-3.

SEM images of G-2 and G-3 indicated that graphene was embedded into the Al@AP particles. The high thermal conductivity of graphene facilitated rapid internal heat transfer, produced from the high-temperature gases produced during combustion, along the graphene surface, thereby rapidly heating the sample in proximity to graphene and forming a heating zone; immediate ignition then occurred when the heated sample reached the ignition temperature. As a result, the maximum pressure and pressure increase rates for G-2 and G-3 were higher than those of G-1. In G-4 and G-5, as mentioned previously, graphene was wrapped around the Al@AP particles. Although the samples near graphene were heated rapidly, the larger specific surface area resulting from a higher graphene content inhibited combustion. Consequently, the maximum pressure and rates of pressure increase for G-4 and G-5 were lower than those of G-3.

### 3.4. Analysis of Combustion Characteristics

**Figure 8:**
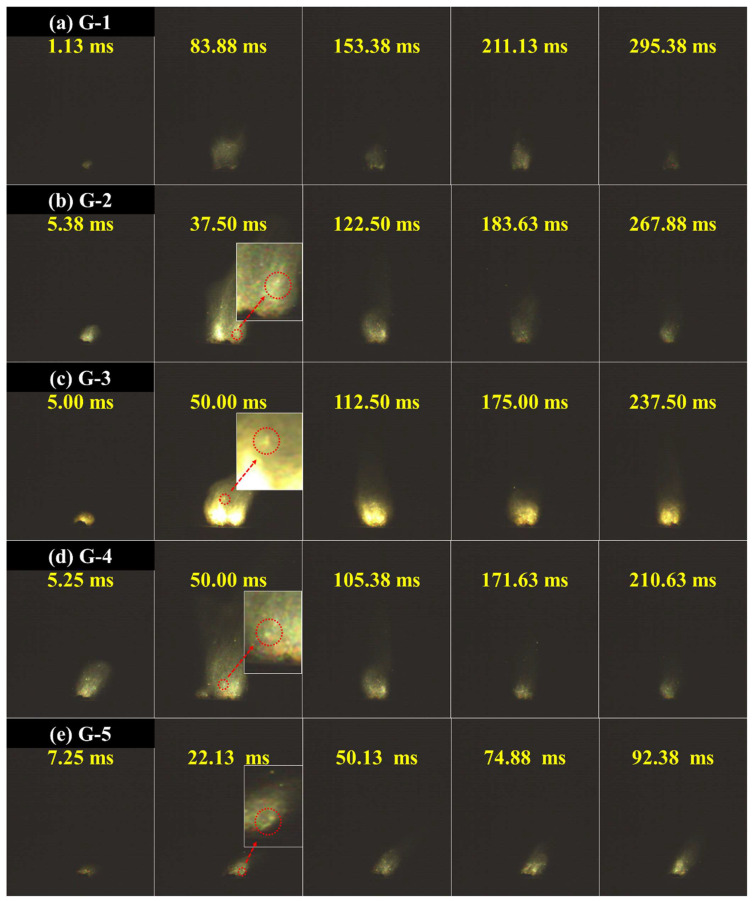
Ignition and combustion images of graphene-doped Al@AP composites with varied doping concentrations.

The ignition and combustion characteristics of the samples were assessed using a laser ignition test. As shown in Figure 8a, the flame intensity gradually increased when G-1 ignited, reaching its peak at 83.88 ms. Subsequently, the flame intensity diminished until reaching a steady state, after which combustion proceeded steadily until the sample was fully consumed. This phenomenon was consistent among all samples and occurred because upon ignition, the flame rapidly spread across the surface of the sample, which increased the combustion area and, in turn, increased flame intensity. Sample surface coverage by the flame was nearly complete as combustion continued, eventually transitioning into laminar burning, in which the flame stabilised until the sample was fully burned.

In addition, the combustion intensity of the samples increased, and the combustion duration decreased with increasing graphene content. This can be attributed to the following: (1) Carbon materials have high emissivity, which increases radiation intensity, and (2) graphene promotes the ignition and combustion of Al in the propellant, increasing the radiation intensity. At the same laser energy input and weight, a lower combustion duration indicated that a higher graphene content resulted in faster propellant combustion. This finding is consistent with the DSC results, which showed that graphene significantly reduced the thermal decomposition temperature of AP. A decrease in AP thermal decomposition temperature decreases the ignition delay and increases the combustion rate of the propellant [31].

Al@AP particle agglomeration was also observed during combustion in G-2 to G-5, where the bright particles represent ignited Al@AP. For G-2 and G-3, typical spherical agglomerates and distinct core-shell structures were observed during combustion. This represented ‘Graphene@(Al@AP)’ combustion, in which graphene-doped Al@AP agglomerates were ejected into the air by combustion gases, where they subsequently burned with graphene, acting as a heat transfer medium. Combined with the FTIR results, it can be inferred that AP combustion was complete at this stage, resulting in a bright flame intensity. For G-4 and G-5, the concentration of typical spherical agglomerated particles decreased with increasing graphene content due to the formation of ‘(Al@AP)@graphene’ agglomerates. Here, graphene enclosed the Al@AP particles into a confined environment where they were unable to burn completely, thereby leading to reduced flame intensity.

### 3.5. Impact and Friction Sensitivities

**Figure 9:**
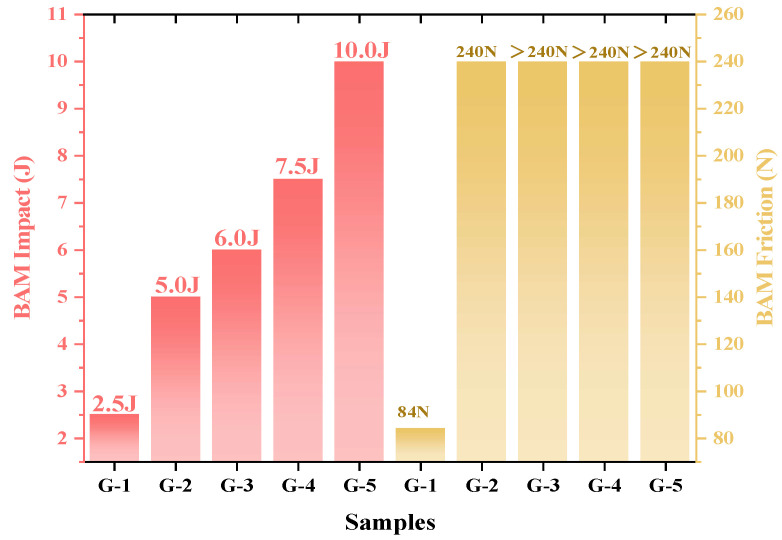
Results of standard BAM impact and friction sensitivity analyses for graphene-doped Al@AP composites with varied doping concentrations.

As shown in Figure 9, the BAM impact and friction sensitivities of G-1 are 2.5 J and 84 N, respectively, which increased to 10.0 J and >240 N when the graphene content was increased to 4.0 wt.%. This improvement is attributed to the excellent lubricating properties of graphene. The layered structure of graphene features weak interlayer bonding forces, resulting in planes that can easily slide over one another. The sliding motion reduces frictional heat generation between energetic materials when external mechanical stimuli are applied, decreasing the formation of hot spots and enhancing the impact and friction performance. Thus, the addition of graphene resulted in a significant improvement in safety performance, indicating that graphene serves as a multifunctional and versatile additive by not only enhancing the combustion performance of Al@AP but also improving its safety characteristics.

### 3.6. Comprehensive Effect of Graphene on Al@AP Performance

**Figure 10:**
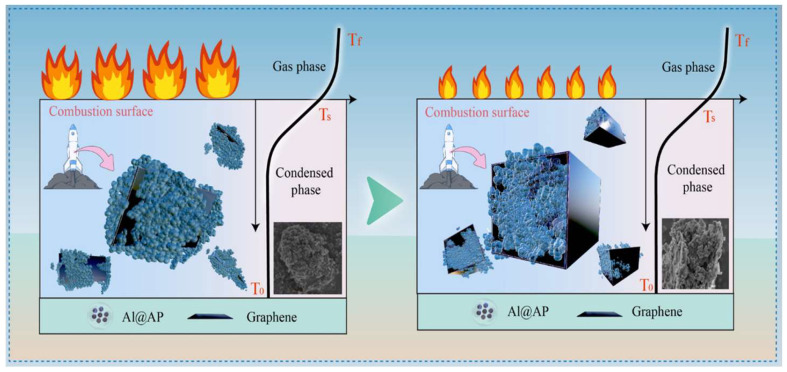
Proposed mechanisms describing the effect of graphene on Al@AP performance.

The experimental results indicate that graphene effectively altered the thermal decomposition, ignition, and combustion characteristics of Al@AP, enhancing its safety performance; the corresponding data is summarised in Table 4, and the comprehensive impact of graphene content on the combustion performance of Al@AP particles is shown in Figure 10. The sample structure was best described as ‘Graphene@(Al@AP)’ when the graphene content was 0.5 wt.% and 1.0 wt.%. Here, the large specific surface area of graphene facilitates electron and heat transfer, increasing the number of reaction sites for AP decomposition and facilitating chemical reactions. In addition, graphene improves the ability to capture and transfer electrons during AP decomposition, accelerating the electron transfer process and promoting the complete decomposition of AP. Furthermore, graphene promotes Al oxidation, improving combustion efficiency and intensity. However, the sample structure was best described as ‘(Al@AP)@Graphene’ when the graphene content was 2.0 and 3.0 wt.%. The large specific surface area of graphene no longer played a promotional role; instead, it enclosed the Al@AP particles, creating a confined environment that inhibited AP decomposition. The mechanism by which graphene influences combustion characteristics can be attributed to its large specific surface area and high thermal conductivity.

As shown in Figure 10, the impact and friction sensitivities of the samples increased with increasing graphene content. The mechanism through which graphene enhances safety performance can be attributed to its excellent lubricating properties. However, despite the continuous improvement in safety performance with the addition of graphene, combustion characteristics could not be optimised simultaneously. In this study, both the combustion and safety performance of the samples were maximised when the graphene content was 1.0 wt.%, thereby achieving a balanced enhancement.

## 4. Conclusions

Al@AP core-shell particles were prepared using an in-situ deposition method with graphene doping to adjust energy release and enhance safety performance. The high electrical and thermal conductivities of graphene, combined with its large specific surface area, facilitated charge transfer during both the low and high-temperature decomposition processes of AP, ultimately decreasing the decomposition temperature. In this study, the addition of 1.0 wt.% graphene effectively increased the rate of pressure increase and combustion characteristics of the Al@AP particles. The lubricating effect of graphene contributed to the excellent safety performance of the samples.

This study provides insight into the relationship between energy release and safety performance in energetic materials, guiding the application of graphene in solid propellants. In future studies, a binder should be added during the formation of solid propellants to investigate the influence of graphene content on their combustion characteristics and safety performance.

## Figures and Tables

**Table 1:** Physicochemical specifications of raw materials.

Material	Specification	Manufacturer
AP	Class II	Liming Chemical Co., Ltd., Luoyang, China
Al	50 nm	Zhongke Detong Technology Co., Ltd.,Beijing, China
Graphene	Less than three layers	Kegong Metallurgical Materials Co., Ltd., Xingtai, China
*N*,*N*-Dimethylformamide (DMF)	ACS	J&K Scientific, Beijing, China
Methanol	HPLC	Beijing Tongguang Fine Chemicals Company, Beijing, China
Ethyl acetate	AR	Beijing Tongguang Fine Chemicals Company, Beijing, China

**Table 2:** Formulation matrix of graphene-modified Al@AP energetic composites.

No.	AP (g)	Al (g)	Graphene (g)
G-1	1.6	0.4	0
G-2	1.6	0.4	0.01
G-3	1.6	0.4	0.02
G-4	1.6	0.4	0.04
G-5	1.6	0.4	0.08

**Table 3:** Peak pressures and rates of pressure increase for graphene-doped Al@AP composites with varied doping concentrations.

Sample	Pressure (kPa)	ΔP/Δt (kPa ms^−1^)
G-1	108.35	5.46
G-2	110.62	11.69
G-3	114.65	13.29
G-4	85.27	12.10
G-5	71.79	6.54

**Table 4:** Representative data for sample thermal decomposition, pressure, and safety performance.

Sample	*T*_LTD_ (°C)	*T*_HTD_ (°C)	Pressure (kPa)	BAM Impact/Friction
G-1	332.77	411.77	108.35	2.5 J/84 N
G-2	310.35	408.15	110.62	5.0 J/240 N
G-3	308.00	410.80	114.65	6.0 J/>240 N
G-4	308.60	403.60	85.27	7.5 J/>240 N
G-5	307.29	395.09	71.79	10.0 J/>240 N

## Data Availability

Data is contained within the article.

## Abbreviations

The following abbreviations are used in this manuscript:

AP	ammonium perchlorate
DMF	Dimethylformamide
LTD	low-temperature decomposition
HTD	high-temperature decomposition
SEM	scanning electron microscopy
TEM	transmission electron microscopy
TG-DSC-FTIR	Thermogravimetric analysis-differential scanning calorimetry-Fourier transform infrared spectroscopy
XRD	X-ray diffraction
